# Supplementary material for: Depression and anxiety in people with cognitive impairment and dementia during the COVID-19 pandemic: Analysis of the English Longitudinal Study of Ageing
Source: PLoS Med. 2023 Apr 24;20(4):e1004162. doi: 10.1371/journal.pmed.1004162 (PMC10124844; doi:10.1371/journal.pmed.1004162)
Supplement: Attachment — Submitted filename: WP4 DepAnx Response to reviewers.docx [file pmed.1004162.s002.docx]

**Submission PMEDICINE-D-22-04054R1:**

**Changes in depression and anxiety among people with cognitive impairment and dementia during the COVID-19 pandemic: Analysis of the English Longitudinal Study of Ageing**

**Response to Reviewers:**

We want to thank the reviewers (and editors) for taking the time to review our manuscript and provide constructive feedback. There are various issues raised across the reviewers, and we hope we have addressed these to satisfaction. We are grateful for the reviewers’ perspectives, and we believe the feedback has helped strengthen the quality of our manuscript. We have provided point-by-point responses to comments below.

Comments from the reviewers:

Reviewer #1: The authors propose a study exploring change in depression and anxiety between before and during the Covid-19 pandemic in three subsamples of people without cognitive impairment, with mild cognitive impairment, and with dementia. The phases in which data was collected and the large sample size make it possible to raise clarity over the results of previous studies on the topic. The analyses and results reported are very robust. I have some minor suggestions for the authors:

-       In the introduction I would like to see some estimations on prevalence of depression and anxiety, MCI and dementia in the second half of life.

- We have added some estimates in lines 18-21 and 23-26.

-       Also, I would like to see discussion of how prevalence of depression and anxiety vary in late middle age, early old age, and advanced old age. I think a limitation of this study may be that all people aged 50+ very analyses together but different age groups may have different evolutions in terms of depression and anxiety.

- We acknowledge that we did not perform any analyses stratified according to age, partly due to the already small proportion of people with dementia in our sample, which would impact results from further segmenting of the sample. We have, however, noted the variation due to age with figures related to depression on lines 27-28.

-       The study aim in the introduction is not very clear in terms of timepoints. Could the authors rephrase the below sentence and be more specific in terms of timepoints: "Using data from the English Longitudinal Study of Ageing (ELSA), we examine changes in depression and anxiety from before the pandemic across two time points during the pandemic with respect to three levels of cognitive function".

- This has been clarified in lines 50-51.

-       In the introduction, when describing the study aims, I would spell out the sociodemographic inequalities investigated.

- This has been clarified in lines 52-53.

-       As the ELSA study started in 2002, where those who remained in the study in 2002 healthier than those who dropped out since 2002? I would comment this.

- It is true that health plays a role in attrition across the years since 2002 for the ELSA sample. However, our study uses data only starting with the 2018/19 Wave 9 data collection, and there have been multiple refreshment samples across the study to maintain representativeness of people aged 50+. We have noted this on lines 59-60.

-       The analyses section is very robust.

- We are very grateful for this positive assessment.

-       How was multimorbidity defined? two or more conditions? Also which conditions were counted? Could the authors please explain this under Table 1 as a note

- We have clarified the construction of the multimorbidity variable, although, rather than including this in Table 1, we have outlined this in the text under the Analytical approach where first mentioned. This can be found on lines 108-111. We did also add (2+ health conditions) to the row in Table 1 for added clarity.

-       For education could the authors explain what consists in low, medium, and high education under Table 1.

- We have clarified the construction of education in Table 1; rather than adding a note, we have simply added text in the table to specify the educational levels associated with low, medium, and high.

-       Minor comment: often the authors use "while" instead of "whereas".

- We thank the reviewer for highlighting this distinction. We identified a number of places in the discussion section where “whereas” was indeed better suited as the subordinating conjunction.

-       In the discussion, first paragraph, could the authors specify the year of Nov/Dec. The same in the remaining paragraphs - years should be specified to ease readability.

- We have added this and ensured that the year features prominently in each paragraph; that is, we have not added it to every mention of a month when the year is clear from the preceding phrase or sentence.

-       Could the authors also discuss the size/clinical relevance of the changes found

- We have added a paragraph referring to this (and the challenges involved) in the discussion on lines 335-345.

-       For those with mild impairment it would be interesting to discuss in the discussion whether the size of the change observed during the pandemic is comparable to the size of the change observed in individuals with mild cognitive impairment over the same period of time in non-pandemic contexts

- We have been unable to find any comparable studies that look at the same measures for depression and anxiety in a population with mild impairment over time.

Reviewer #2: The manuscript is a study about changes in depression and anxiety among people with cognitive impairment during COVID-19. I think the manuscript deserve publication in this journal however some changes are necessary.

- I think the N sample size needs to be in the abstract, the methods and Findings section and in Table 1.

- The sample sizes used in this paper were highlighted in the Results section after Table 1; this can be found in the revised manuscript lines 144-146. We have added the key sample size to the abstract and added absolute numbers to Table 1.

- I would like to know how the authors handle the multiple comparisons made in the study and if they have made corrections for that. If not I would like to know the justification for that.

- We would like to clarify that multiple comparisons were not undertaken, therefore adjustment like Bonferroni was not needed. The reviewer may be referring to the difference in the single-item anxiety measure and the GAD-7 measure; however, this comparison is only illustrative, since the measures are different. With GAD-7 unmeasured before the pandemic, we include both analyses to show 1) the more robust measure of anxiety during the pandemic and 2) how anxiety changed from before the pandemic based on the available single-item.

- In the result section it is only indicated the value in anxiety and depresion in differents temporal points (and CI) but not the value of change or a value about effect size with the CI or p value. I think this measure are needed. At least I would like to know why this measures are not reported.

- In our study, we have estimated depression and anxiety scores across time and cognitive function, with the intention to understand the dynamics of change over time. There are challenges in putting a value on any magnitude of change in these scales, and we have added a paragraph referring to this (and the challenges involved) in the discussion on lines 335-345.

Thank you for the opportunity to review the manuscript

Kind regards

Reviewer #3: This is a well-conducted study on the changes in depression and anxiety among people with cognitive impairment and dementia during the COVID-19 pandemic using the ELSA dataset. The study design, datasets, statistical methods and analyses, and presentation (tables and figures) and interpretation of the results are mostly adequate and of a good standard. However, still a few issues needing attention.

1) There is no mention of statistical methods at all in the abstract.

- This is now included.

2) Sample size was not clearly mentioned. Can the authors please make the sample size clear at each survey throughout the manuscript including text, tables and figures?

- The sample sizes used in this paper were highlighted in the Results section after Table 1; this can be found in the revised manuscript lines 144-146. All analyses have a sample size of 5,286 except those looking at the GAD-7 anxiety score as the outcome, where it is reduced by five to 5,281.

3) Table 1. Count and percentage are both needed instead of just the percentage.

- We have added absolute numbers (counts) to Table 1.

4) Can authors please confirm whether the estimates in Figure 1 to 5 were adjusted for all the confounders as described in the methods secion Page 13?

- Yes, that is correct, all models and analyses reported in the manuscript included full adjustment. We have added a sentence to this effect on lines 146-148.

5) Rating scales. There are so many rating scales for depression and anxiety, up to around 20 including the ones like HADS and etc. Can the authors go a bit further in the discussion on why and justifications of using CES-D or GAD-7 as compared to other widely used scales? Pros and cons?

- ELSA is part of the family of the HRS ageing cohort studies, and as such was initiated to be comparable with HRS. Therefore the depression scale that the study adopted was that already used in HRS, the GAD-7 was adopted for the same reason. We have used GAD-7 and CES-D because these are the scales included in the ELSA survey. We have added text and citations in support of their use in the discussion, on lines 324-329.

6) In the abstract, it says "Using a clinical cutoff for likely depression (CES-D≥4), we found statistically significant increases in the probability of likely clinical depression between 2018/19 and Nov/Dec 2020 for those with no impairment (0.110 (0.099-0.120) to 0.206 (0.191-0.222)) and those with mild impairment (0.139 (0.120-0.159) to 0.234 (0.204-0.263))". I am not sure what is meant by 'probability of likely clinical depression'. By a cutoff, we then can identify a proportion of patients with likely depression, rather than probability, is that right?

- Yes, that is correct; the use of the cutoff indicates likely clinical depression, as a formal diagnosis would require professional consultation. In the case of our analyses using the cutoff, we have a binary measure identifying likely cases. From the mixed-effects logistic regression models, we have plotted estimates of the average predicted probability of likely cases; we thus opted for the phrase “probability of likely clinical depression”.

Requests from the editors:

Please include continuous line numbering throughout your manuscript.

- These are now added from the start of the main text.

Abstract Methods and Findings:

* Please include the study design and number of participants.

- These have been added.

* Please include the important covariates that are adjusted for in the analyses.

- These have been added.

* In the last sentence of the Abstract Methods and Findings section, please describe the main limitation(s) of the study's methodology.

- This has been added.

At this stage, we ask that you include a short, non-technical Author Summary of your research to make findings accessible to a wide audience that includes both scientists and non-scientists. The Author Summary should immediately follow the Abstract in your revised manuscript. This text is subject to editorial change and should be distinct from the scientific abstract. Please see our author guidelines for more information: <https://eur01.safelinks.protection.outlook.com/?url=https%3A%2F%2Fjournals.plos.org%2Fplosmedicine%2Fs%2Frevising-your-manuscript%23loc-author-summary&data=05%7C01%7Cb.beach%40ucl.ac.uk%7Ca37c2343fea64404948508db0dd6916b%7C1faf88fea9984c5b93c9210a11d9a5c2%7C0%7C0%7C638118987437163377%7CUnknown%7CTWFpbGZsb3d8eyJWIjoiMC4wLjAwMDAiLCJQIjoiV2luMzIiLCJBTiI6Ik1haWwiLCJXVCI6Mn0%3D%7C3000%7C%7C%7C&sdata=O6u03pt%2BrAqjyO%2FVlNaGRGw00ZFTjWG2uRzM0c1%2F4XA%3D&reserved=0>

- We have added the author summary after the abstract.

Please place citations in square brackets.

- This has been done.

Please ensure that the study is reported according to the STROBE guideline, and include the completed STROBE checklist as Supporting Information.  Please add the following statement, or similar, to the Methods: "This study is reported as per the Strengthening the Reporting of Observational Studies in Epidemiology (STROBE) guideline (S1 Checklist)."

- This has been included in lines 135-136.

The STROBE guideline can be found here: <https://eur01.safelinks.protection.outlook.com/?url=http%3A%2F%2Fwww.equator-network.org%2Freporting-guidelines%2Fstrobe%2F&data=05%7C01%7Cb.beach%40ucl.ac.uk%7Ca37c2343fea64404948508db0dd6916b%7C1faf88fea9984c5b93c9210a11d9a5c2%7C0%7C0%7C638118987437163377%7CUnknown%7CTWFpbGZsb3d8eyJWIjoiMC4wLjAwMDAiLCJQIjoiV2luMzIiLCJBTiI6Ik1haWwiLCJXVCI6Mn0%3D%7C3000%7C%7C%7C&sdata=d1gM8WjpnEOcDQqdootRnNjVSXbEMVSkQclca4Qhznc%3D&reserved=0>

When completing the checklist, please use section and paragraph numbers, rather than page numbers.

- This has been completed and the checklist provided.

Did your study have a prospective protocol or analysis plan? Please state this (either way) early in the Methods section.

a) If a prospective analysis plan (from your funding proposal, IRB or other ethics committee submission, study protocol, or other planning document written before analyzing the data) was used in designing the study, please include the relevant prospectively written document with your revised manuscript as a Supporting Information file to be published alongside your study, and cite it in the Methods section. A legend for this file should be included at the end of your manuscript.

b) If no such document exists, please make sure that the Methods section transparently describes when analyses were planned, and when/why any data-driven changes to analyses took place.

c) In either case, changes in the analysis-- including those made in response to peer review comments-- should be identified as such in the Methods section of the paper, with rationale.

- We did not have a prospective analysis plan. The analyses were planned at the conception stage, i.e. in the grant application. We have added a sentence to address option b) above on lines 97-98.

For all observational studies, in the manuscript text, please indicate: (1) the specific hypotheses you intended to test, (2) the analytical methods by which you planned to test them, (3) the analyses you actually performed, and (4) when reported analyses differ from those that were planned, transparent explanations for differences that affect the reliability of the study's results. If a reported analysis was performed based on an interesting but unanticipated pattern in the data, please be clear that the analysis was data-driven.

- This has been addressed in added text on lines 98-104.

Please include the numerators and denominators in Table 1.

- We have included the numerators for each item in an additional column. The figure for the denominator is indicated in the heading of the column.

Figures 1-5: Please begin the y-axes at zero or include a break in the axes.

- We have scaled the y-axes for the figures to facilitate visual inspection for the reader, especially given the closeness of the tails for the confidence intervals. We believe this is more effective given that the values on the axes are clearly indicated. We would add a break on the axes between 0 and the starting point, but this is not a feature available in Stata, where we have generated the graphs. We have added text to highlight the restricted axis scales in lines 147-148.

The terms gender and sex are not interchangeable (as discussed in <https://eur01.safelinks.protection.outlook.com/?url=https%3A%2F%2Fwww.who.int%2Fhealth-topics%2Fgender&data=05%7C01%7Cb.beach%40ucl.ac.uk%7Ca37c2343fea64404948508db0dd6916b%7C1faf88fea9984c5b93c9210a11d9a5c2%7C0%7C0%7C638118987437163377%7CUnknown%7CTWFpbGZsb3d8eyJWIjoiMC4wLjAwMDAiLCJQIjoiV2luMzIiLCJBTiI6Ik1haWwiLCJXVCI6Mn0%3D%7C3000%7C%7C%7C&sdata=ub%2B%2BxFeyZWumovgbvggLA46qjWo0fc%2F2Ih1yoSTIhk0%3D&reserved=0>); please confirm that gender is the appropriate term for your study.

- We are glad that the editors highlight this important distinction. We can confirm that gender is the correct term for our study. We do not focus on biological mechanisms more relevant for sex; instead, our study is more interested in the social processes related to gender that might generate differential experiences in terms of mental health and access to services.

Some of the content in your Results would be better placed in the Discussion (any interpretation, e.g., ‘In other words, it appears that being in the poorest third…’).

- We did not find any clear examples of interpretation of results that we believe should be moved to improve the discussion. From our perspective, the example provided above was included simply to help clarify the numerical results from the interaction in terms of the conceptual meaning. We would be happy to delete this sentence.

Please consider tabulating your comparisons to facilitate interpretation for the reader.

- We feel the best representation of our results is presenting them in the graphical figures included in the manuscript. We also acknowledge guidelines to avoid reporting results in both graphic and tabular formats.

Please remove funding details from your Acknowledgements and instead ensure they are included in your Financial Disclosure in the Submission Form.

- This has been removed from the manuscript.

Please remove the Author Contributions, Competing Interests, Data Availability and Disclaimer sections from the main text and ensure all relevant details are captured in the appropriate part of the Submission Form.

- These sections have been removed from the manuscript.

Please relocate the Ethical Approval Statement to the Methods section and confirm whether you sought advice from an Institutional Review Board.

- This has been corrected, and the ethics statement can be found on lines 66-67.

For Internet sources in the References, please include the date accessed.

- This has been corrected.
