# Supplementary material for: Depression and anxiety in people with cognitive impairment and dementia during the COVID-19 pandemic: Analysis of the English Longitudinal Study of Ageing
Source: PLoS Med. 2023 Apr 24;20(4):e1004162. doi: 10.1371/journal.pmed.1004162 (PMC10124844; doi:10.1371/journal.pmed.1004162)
Supplement: Attachment — Submitted filename: R2 response to editors.docx [file pmed.1004162.s003.docx]

**Submission PMEDICINE-D-22-04054R2:**

**Depression and anxiety in people with cognitive impairment and dementia during the COVID-19 pandemic: Analysis of the English Longitudinal Study of Ageing**

**Response to Reviewers:**

We again thank the reviewers for their feedback. We further thank the editors for the additional points to progress publication of our manuscript. Please see point-by-point responses outlined below.

Please update your title to ‘Depression and anxiety in people with cognitive impairment and dementia during the COVID-19 pandemic: Analysis of the English Longitudinal Study of Ageing’.

- Done.

‘All figures reported below reflect the full model, adjusted for all available covariates’ – please include this information in the legends of Figures 1-5.

- Done.

Author Summary:

* Include the headline numbers from the study, such as the sample size and key findings under the question ‘What did the researchers do and find’.

- Key figures have been added to the second and third bullet points.

* Typo in bullet point 2 under the question ‘What do these findings mean’.

- This has been amended.

Please update the final paragraph of your introduction to reflect the hypotheses described at lines 97-104 (the abstract, author summary, and discussion ought to be similarly updated).

- The final paragraph of the introduction and the first parts of the abstract, the author summary, and the discussion now include text directly reflecting on these hypotheses.

At line 202, the term "trend" is used to refer to a nonsignificant P value. The term trend should be used only when the test for trend has been conducted. Please revise accordingly.

- This has been revised to “trajectory”.

I feel that the results relating to inequalities in mental health and cognitive function ought to be acknowledged, at least briefly, in the abstract and author summaries (this can be a single sentence summary).

- The abstract has been further edited to incorporate the statement, “Wealth and education appeared to be stronger drivers for depression and anxiety, respectively, than cognitive impairment.” An additional bullet point to this effect was also included in the author summary.

Reference 9 contains an asterisk, the meaning of which is unclear.

- The asterisk appears as part of the title of the article and associated citations, though it seems to refer to the article’s submission date. This has been amended.

Please check the panel labels in Figure 4 – it looks as though some symbols have been replaced with a question mark.

- This has been corrected.
