# Supplementary material for: Depression and anxiety in people with cognitive impairment and dementia during the COVID-19 pandemic: Analysis of the English Longitudinal Study of Ageing
Source: PLoS Med. 2023 Apr 24;20(4):e1004162. doi: 10.1371/journal.pmed.1004162 (PMC10124844; doi:10.1371/journal.pmed.1004162)
Supplement: Attachment — Submitted filename: R3 response to editors.docx [file pmed.1004162.s004.docx]

**Submission PMEDICINE-D-22-04054R3:**

**Depression and anxiety in people with cognitive impairment and dementia during the COVID-19 pandemic: Analysis of the English Longitudinal Study of Ageing**

**Response to Reviewers:**

We again thank the editors for allowing to revise our manuscript for publication. Please see point-by-point responses outlined below.

I feel there is still some confusion around the presentation of the hypotheses. It is essential that the manuscript clearly and consistently describes the hypotheses you intended to test and the analytical methods used to test them.

- We apologise for confusing the presentation of the hypotheses. We have revised the text around the hypotheses in the abstract, author summary, and main text (introduction and analytical approach sections). Hopefully these revisions offer greater clarity and consistency. In doing so, we have taken on board the further points below.

The abstract and author summary both do not mention that the investigation was intended to investigate ‘how associations would vary across key sources of socioeconomic inequality’ (Methods), nor do they provide quantitative data to support the statement ‘Wealth and education appeared to be stronger drivers for depression and anxiety, respectively, than cognitive impairment’.

- Text to this effect has been added to both the abstract and author summary. We also provide quantitative data in the form of one example (wealth and depression in June/July 2020). Adding this (and other requested revisions) have required substantial edits to the abstract, but we have retained all information and key points within the word count. We hope the one example on the inequalities is sufficient.

Related to the above, I feel the description of this hypothesis in the Introduction (‘We also investigate whether key sociodemographic inequalities related to wealth, education, geographic region, and multimorbidity are linked to patterns in outcomes’) differs from that in the Methods. Please clarify exactly what hypothesis you intended to test and provide a harmonised description throughout the manuscript.

- We have clarified this and provided harmonised text.

Your study is observational and therefore causality cannot be inferred. Please adjust the way your hypotheses are worded to reflect this in the Abstract, Author Summary, and throughout. For example, I feel that the sentence ‘This study addresses such gaps in the evidence base to test whether depression and anxiety among older people was worse with increasing cognitive impairment and worsened from before to during the COVID-19 pandemic’ would be better presented as ‘This study investigated whether changes in depression and anxiety in older people during the COVID-19 pandemic were associated with level of cognitive impairment’.

- We are grateful for the suggested rephrasing to avoid implying causality. We have incorporated this as part of the revisions.
